# Behavioral and Neuroanatomical Abnormalities in Pleiotrophin Knockout Mice

**DOI:** 10.1371/journal.pone.0100597

**Published:** 2014-07-07

**Authors:** Jason W. Krellman, Henry H. Ruiz, Veronica A. Marciano, Bracha Mondrow, Susan D. Croll

**Affiliations:** 1 Neuropsychology Doctoral Subprogram, Graduate Center of the City University of New York, New York, New York, United States of America; 2 Icahn School of Medicine at Mount Sinai, New York, New York, United States of America; 3 Department of Psychology, Queens College of the City University of New York, Flushing, New York, United States of America; 4 Regeneron Pharmaceuticals, Tarrytown, New York, United States of America; Roma Tre University, Italy

## Abstract

Pleiotrophin (PTN) is an extracellular matrix-associated protein with neurotrophic and neuroprotective effects that is involved in a variety of neurodevelopmental processes. Data regarding the cognitive-behavioral and neuroanatomical phenotype of pleiotrophin knockout (KO) mice is limited. The purpose of this study was to more fully characterize this phenotype, with emphasis on the domains of learning and memory, cognitive-behavioral flexibility, exploratory behavior and anxiety, social behavior, and the neuronal and vascular microstructure of the lateral entorhinal cortex (EC). PTN KOs exhibited cognitive rigidity, heightened anxiety, behavioral reticence in novel contexts and novel social interactions suggestive of neophobia, and lamina-specific decreases in neuronal area and increases in neuronal density in the lateral EC. Initial learning of spatial and other associative tasks, as well as vascular density in the lateral EC, was normal in the KOs. These data suggest that the absence of PTN *in vivo* is associated with disruption of specific cognitive and affective processes, raising the possibility that further study of PTN KOs might have implications for the study of human disorders with similar features.

## Introduction

Pleiotrophin (PTN), also known as heparin-binding neurotrophic factor (HBNF) and heparin-binding growth-associated molecule (HB-GAM), is an extracellular matrix-associated protein implicated in a variety of processes integral to mammalian nervous system development [1], including mitogenesis and neurite outgrowth [2]–[6], cellular differentiation [7], [8], arrest of cellular proliferation [9], cell motility [10], early presynaptic [11] and postsynaptic specialization [12], and vasculogenesis [13], [14]. Pleiotrophin also exerts post-developmental neurotrophic and neuroprotective effects on nigrostriatal dopaminergic neurons [15]–[17], on distal sciatic nerves and spinal motor neurons after denervation [18], and on microglia after experimentally-induced ischemic/reperfusion injury [19].

PTN expression in the normal nervous system declines in most regions as constitutive developmental processes approach completion [6], but expression persists in specific neuronal populations, particularly in pyramidal neurons of the CA1 field of the hippocampus [20], [21], where PTN might facilitate neuronal repair after ischemic injury [20]. In addition, induction of hippocampal long-term potentiation (LTP) results in up-regulation of PTN in CA1 [22], and application of exogenous PTN inhibits early LTP in CA1 glutamatergic synapses [22], [23]. These data suggest that PTN is an inhibitory modulator of hippocampal LTP. Indeed, mice null for the PTN gene *Ptn* show a lowered threshold for LTP in slice despite normal basal excitatory synaptic transmission [24], and LTP is significantly attenuated in animals over-expressing PTN [25].

Hippocampal LTP is a putative neurobiological correlate of learning and memory [26], suggesting that PTN knockouts (KOs) might show abnormalities in these and other cognitive and/or behavioral domains. However, neurobehavioral data in PTN KOs are limited. Pavlov et al. [25] reported increased anxiety and a subtle impairment in spatial information acquisition in PTN KOs, while others have reported findings suggestive of enhanced learning and memory, such as prolonged maintenance of recognition memory for spatial information [27] and increased persistence of drug-seeking behavior after discontinuation of amphetamine administration [28]. Neuroanatomical studies of PTN KOs are similarly limited, with only increases in neuronal density in frontal and parietal cortices reported previously [9].

The purpose of the current study was to more fully characterize the neurobehavioral and neuroanatomical phenotype of PTN KOs, with emphasis on the domains of learning and memory, cognitive-behavioral flexibility, exploratory behavior and anxiety, and social behavior. In addition, we conducted a structural analysis of neurons and vasculature in the deep layers of the KOs' lateral entorhinal cortex (EC), as this area has been implicated in both learning and memory and affective responses such as those previously shown to be abnormal in these animals [25].

## Methods

### Animals

Four cohorts of mice (on a 50% 129; 50% C57B1/6 inbred strain mix) null for PTN (*Ptn*−/−) were used from a line created as previously described [24]. Controls for each cohort were either wild type littermates (2 cohorts) or parallel-bred F2 mice from F1 homozygous siblings (2 cohorts). All behavioral tests and neuroanatomical assessments were conducted on at least 2 cohorts, and the order of behavioral tests was counterbalanced among cohorts to control for order effects. Preliminary analyses were conducted to confirm no interaction between the effects of genotype and cohort before data from different cohorts were combined. Animals were housed in small groups of 2–5 siblings per cage (equivalently between genotypes). Three [2 wild type (WT) and 1 KO] animals that repeatedly demonstrated aggressive and injurious behavior toward cage mates were housed individually. All animals were provided food and water *ad libitum*. The colony room was maintained at 23 degrees Celsius on a 12 hour light-dark cycle (lights off at 19:00). The current study used 24 PTN KOs and 21 wild type (WT) mice balanced for sex.

This study was carried out in strict accordance with the recommendations in the Guide for the Care and Use of Laboratory Animals of the National Institutes of Health. All procedures were conducted with approval from and in strict compliance with the animal welfare policies of the Institutional Animal Care and Use Committee of Queens College of the City University of New York. All efforts were made to minimize animals' suffering.

### Behavioral Testing Procedures

Animals were approximately 4–5 months old during behavioral testing. Individual animals were excluded from testing in the event of either apparent illness or injury (e.g. from skirmishes with cage mates). Animals were excluded from testing in the open field (1/9 KOs and 3/7 WTs) and social approach (1/9 KOs and 1/8 WTs) paradigms. Chi Square analyses revealed no significant difference in the frequency of exclusion between the genotypes in either paradigm (open field: χ^2^ = 1.63, *p* = .29; social approach: χ^2^ = .01, *p* = .93). Inter-test intervals (“washout periods”) varied between 7 and 14 days, which has been deemed adequate to reduce the effect of prior test experience on performance [29].

Animals were allowed 60 minutes to acclimate to the testing room in their home cage prior to all behavioral testing. All behavioral quantification and analyses were conducted by an experimenter blind to animal genotype. General physical (e.g. weight) and behavioral (e.g. home cage behavior) assessments were made as described in Crawley [30]. The behavioral testing paradigms used have been validated for use with rodents [30]–[32].

### Learning and Memory

Learning and memory was assessed using the Morris water maze as described previously [33]. Briefly, animals' latency to escape the maze onto a hidden platform was measured in 3 trials per day (a “trial block”), separated by a 30 second inter-trial interval, over 4, 5 or 8 days depending on rate of learning (animals were trained until WTs' latency to escape was 10 seconds or less throughout a single trial block). Data were analyzed as median latency to escape per block of 3 trials. Animals were tested using 2 distinct versions of the paradigm: *spatial* and *cued*. In the spatial version, the location of the hidden escape platform was constant across trials so that animals needed to use extramaze spatial cues to locate the platform. In the cued version, the platform location was varied among trial blocks and a clear/black striped acetate sheet measuring 30×10 cm was affixed to the inside of the maze adjacent to the platform as a distinct visual cue so that animals learned to associate the cue with the location of the escape platform. In both maze variations, animals were led to the platform by hand if they had not located the platform after 1 minute. For both versions, animals were returned to the maze 24 hours after the last acquisition trial with the platform removed and were tracked for 30 seconds as a test of retention of the platform's location. In the spatial version, retention was operationalized as time spent in the quadrant that formerly contained the platform. In the cued version, retention was operationalized as time animals spent in a quadrant containing the striped acetate. Animals that have learned successfully are expected to spend more time in the quadrant that should have contained the platform. Variation in the number of trial blocks required for animals to find the platform in 10 seconds or less was due to the fact that animals learned the cued version of the maze and the first version of the maze to which they are exposed more rapidly than the spatial or second version of the maze.

### Cognitive-Behavioral Flexibility

Cognitive-behavioral flexibility was assessed using the Y-maze, a spontaneous alternation task, as previously described [30], and also by exposing animals to 2 versions of the Morris water maze in 2 different sequences to determine the extent to which animals' learning was affected by recent exposure to a previous version of the maze.

### Y-Maze

Animals were placed in the lower arm of a Y-shaped maze and allowed to explore until they traversed at least half the length of 1 of the maze's other 2 arms. Once an animal reached such a point, it was removed from the apparatus and then returned to the lower arm after a 3 second rest. The procedure was repeated over 11 trials for each animal. For each trial, the animal's choice of arm (left or right, as defined by the animal traversing at least half the length of the arm) was recorded. Animals are expected to spontaneously alternate their choice of arm. Animals' time to make an arm choice was also recorded. The proportion of identical, consecutive arm choices (perseverative arm choices) relative to total number of arm choices made was also recorded. Testing was discontinued for any animal that did not “choose” an arm within 5 minutes in two consecutive trials. Animals failing to produce data for four consecutive trials were excluded from data analysis.

### Morris Water Maze

Animals were tested in 2 sequences for the water maze to evaluate their ability to adapt to changing learning contingencies, or cognitive-behavioral flexibility. For 2 of 4 cohorts, animals were tested in the spatial version of the water maze and then were tested in the cued version of the water maze 2 weeks later. Eight weeks after this first sequence of testing, animals were tested in the cued version and then in the spatial version (with new goal quadrants). The other 2 of 4 cohorts were exposed to the 2 versions of the maze in the opposite order: cued then spatial.

### Exploratory Behavior and Anxiety

Exploratory behavior and anxiety were assessed using the open field and elevated-plus maze paradigms as described previously [30].

### Open Field

Animals were placed in the center grid of the apparatus and allowed to freely explore the maze for 10 minutes. Animals were tested in the open field again 24 hours later to measure habituation. In each trial, animals' latencies to groom and to exit the center grid were recorded. These measures assessed anxiety-like behavior because grooming has been associated with compulsive anxiety, and rodents' natural inclination is to hug the walls of an apparatus rather than stand exposed in the center [30]. In addition, the number of grooming acts was recorded as a measure of animals' inclination to engage in repetitive stereotypical motor behavior, and the animals' total number of grid crossings was recorded as a measure of general locomotor and exploratory behavior.

### Elevated Plus Maze

Animals were placed on the center platform of a plus-shaped maze and allowed to freely explore the maze for 5 minutes. The number of seconds animals spent on the platform and in the 2 open and 2 closed arms was recorded. Increased time spent in the closed arms is considered indicative of heightened anxiety. The number of open and closed arm entries made by animals was recorded as a measure of motor activity and exploratory behavior. Animals' latency to first arm entry was recorded as a measure of behavioral initiation.

### Social Behavior

Social behavior was assessed using the social approach apparatus, which has 3 chambers separated by doors, to administer the tests of sociability and social novelty as described by Moy et al. [34].

### Sociability Test

Animals were placed in the center chamber with the doors to the outer chambers closed and were allowed to explore for 5 minutes, after which a novel WT mouse (stranger 1) was placed in 1 of the 2 adjoining chambers, counterbalanced for side. The stranger was enclosed in a cylinder of wire mesh 11 cm high×10.5 cm in diameter with bars spaced 1 cm apart to permit nose contact between animals. The stranger had been acclimated to the cylinder for 5 minutes prior to its introduction to the apparatus. An identical but empty wire mesh cylinder was placed in the adjoining, empty chamber to balance the novelty of the physical stimuli in the chambers. The doors to the outer chambers were then opened and test animals were allowed to freely explore the entire apparatus for 10 minutes. The number of entries and amount of time animals spent in the center, empty, and stranger 1 chambers were recorded. Animals typically demonstrate a preference for social interaction as evidenced by increased time spent in the stranger 1 chamber relative to the center and empty chambers.

### Social Novelty Test

Immediately following the sociability test, animals were briefly returned to the center of the apparatus and the doors to the adjoining chambers were closed. A second novel WT mouse (stranger 2), also enclosed in and acclimated to a wire mesh cylinder, was placed in the previously empty chamber. The doors to the adjoining chambers were then re-opened and animals were allowed 10 minutes to freely explore the entire apparatus. To assess animals' preference for interaction with stranger 2 over stranger 1, the number of entries and amount of time animals spent in each stranger chamber was recorded, as was the amount of time animals spent in the center chamber. Animals typically spend more time in the chamber housing the more novel stranger 2 than in the center chamber or the chamber housing the less novel stranger 1.

### Histology

At approximately 6–7 months of age, animals were deeply anesthetized and overdosed with chloral hydrate-pentobarbital, transcardially exsanguinated with heparinized isotonic (0.9%) saline, and perfusion fixed with 4% paraformaldehyde, first in low pH buffer (acetate, 100 ml) and then in high pH buffer (borate, 100 ml). After fixation, brains were removed and placed in 30% sucrose in borate buffer for 3 to 7 days at 4°C. Mouse brains were then frozen and sectioned at 30 µm on the coronal plane using a sliding microtome (Leica Biosystems, Inc., Buffalo Grove, IL). Frozen sections were stored at −20°C in cryoprotectant solution as in Watson, Wiegand, Clogh & Hoffman [35].

### Analysis of Lateral Entorhinal Cortical Neurons

Brain sections were mounted on gelatin-coated slides, stained with cresyl violet, and then placed under an Olympus BX51 microscope (Olympus Inc., Center Valley, PA). The Neurolucida image analysis software (version 8.001, MicroBrightfield BioSciences, Williston, VT) was used to manually trace neurons and obtain neuronal area in squared micrometers in layers IV and V of the lateral EC. Approximately 10–20 pseudo-randomly selected cells were measured per frame bilaterally (approximately 20–40 cells per animal). Inter-neuronal distance was measured using Image J software (National Institutes of Health, Bethesda, MD). A standard number of neurons (approximately 20 per animal) met by points on a stereology grid were randomly chosen for measurement. Inter-neuronal distance was defined as the distance in micrometers between the center of a randomly chosen neuron to the center of its closest neighboring neuron in the same plane of focus. To assess lateral EC thickness, 3 images per animal were acquired by a Diagnostic Instruments 320 video camera (Diagnostic Instruments, Sterling Heights, MI) attached to a Nikon microscope (Morell Instruments, Melville, NY). Images were transferred to a computer using Spot Digital Imaging software (Diagnostic Instruments, Sterling Heights, MI) and analyzed using Image J software (National Institutes of Health, Bethesda, MD). All measures were taken by experimenters blind to animal genotype.

### Analysis of Entorhinal Cortical Vasculature

Analysis of cerebral vasculature was conducted as previously described [36]. Briefly, mouse brains were stained with alpha collagen-IV (primary antibody: rabbit-anti-mouse alpha collagen-IV, 1∶500, Chemicon, Temecula, CA; secondary antibody: goat anti-rabbit, 1∶500, R & D Systems, Minneapolis, MN) to visualize cerebral vasculature basement membranes. Stained sections were then mounted on gelatin-coated slides, which were dehydrated via immersion in ethyl alcohol and cover-slipped after subsequent immersion in xylenes.

Stereology to assess vascular diameter was performed on alpha collagen IV-stained brain sections after acquisition of 3 images per region per animal by a video camera (Diagnostic Instruments, Sterling Heights, MI) attached to a Nikon microscope (Morell Instruments, Melville, NY). Images were transferred to a computer using Spot Digital Imaging software (Diagnostic Instruments, Sterling Heights, MI) and analyzed using Image J software (National Institutes of Health, Bethesda, MD).

Vascular density was assessed via point-count stereology as previously described [36] on images captured from layers V–VI of the lateral EC. All points that fell on a vessel were counted. Vascular density was defined as the proportion of points falling on alpha collagen IV-positive tissue across all 3 images to the total number of points in the grid multiplied by 3. To assess vascular diameter, a standard number of vessels (approximately 20–30 per animal) met by points on a stereology grid were randomly chosen for measurement, which was performed using Image J software. All measures were taken by experimenters blind to animal genotype.

### Data Analysis

Statistical analyses were conducted using the Statistical Package for the Social Sciences (SPSS) software version 20.0 (SPSS Inc., Chicago, IL). Analyses involving 2 levels of a single factor (e.g. genotype) and 1 dependent measure were analyzed using t-tests. Analyses involving more than 2 levels of a factor were conducted using analysis of variance (ANOVA), one-way or factorial where appropriate, followed by post hoc Tukey tests to probe significant main effects with multiple levels or significant interaction effects in mixed designs. Chi square analysis was used to determine distributions of animals between categorical variables. Pearson's correlation coefficients were calculated to assess the association between variables. Outliers, defined *a priori* as animals yielding values ≥2 standard deviations from the group mean, were removed from analyses. A *p*<.05 was considered statistically significant for all analyses. Data in figures are presented as means and standard error of the means.

## Results

### General Physical and Behavioral Assessment

PTN KOs' gross reflexes, body weight, balance, grip strength, climbing behavior, home cage behavior, and ease of handling were all comparable to WTs (data not shown).

### Learning and Memory

#### Morris water maze

**Figure 1:**
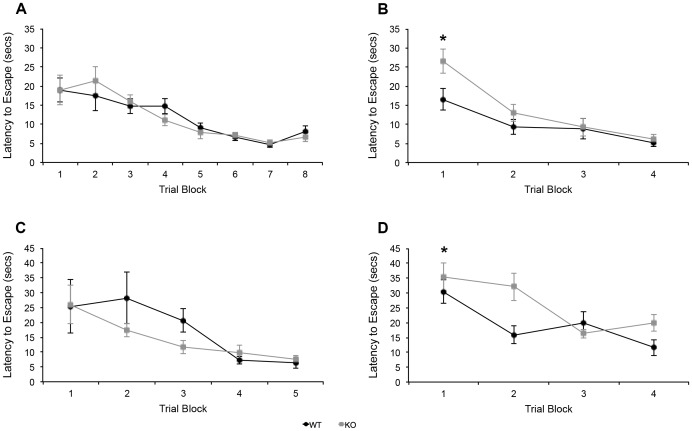
Latencies to escape the Morris water maze for PTN KOs (n = 13) and WTs (n = 13). (a) Spatial version, first maze; (b) cued version, first maze; (c) cued version, second maze; (d) spatial version, second maze. *p = <0.05.

For the first sequence of water maze testing (spatial then cued), both KOs and WTs demonstrated a significant decrease in latency to escape the spatial version across trials (*F* (25, 175) = 17.51, *p* = .000). Similarly, the genotypes did not differ significantly in mean latency to escape across trials (*F* (1, 18) = 2.97, *p* = .998, Figure 1a), suggesting that the KOs learned the first (spatial) version in which they were tested comparably to WTs. In the subsequent cued version, both KOs and WTs again showed a significant decrease in latency to escape across trials (*F* (25, 175) = 8.45, *p* = .000, Figure 1b), but the KOs had a significantly greater mean latency to escape across trials as compared to WTs (*F* (1, 18) = 4.81, *p* = .041), suggesting delayed learning of the second (cued) version in the KOs.

For the second sequence of water maze testing (cued then spatial), KOs and WTs both demonstrated a significant decrease in latency to escape across trials (*F* (14, 140) = 3.58, *p* = .000). The genotypes did not differ significantly in mean latency to escape across trials (*F* (1, 10) = .290, *p* = .602, Figure 1c), suggesting that the KOs learned the first task as quickly as WTs. In addition, this finding suggests that the KOs displayed no impairment in cued water maze learning that would account for their delayed learning of the cued water maze in the first sequence. In the subsequent spatial version, both KOs and WTs showed a significant decrease in latency to escape across trials (*F* (3, 45) = 13.04, *p* = .000, Figure 1d), but the KOs demonstrated a significantly greater mean latency to escape across trials (*F* (1, 15) = 3.96, *p* = .012), indicating that the KOs were once again impaired on the second learning task to which they were exposed, regardless of whether that task was the spatial or cued version of the water maze. In addition, the genotype by trial interaction was significant (*F* (3, 45) = 3.775, *p* = .017). KOs and WTs performed similarly in all retention trials in the water maze (p>.05, data not shown), as evidenced by the genotypes spending a statistically comparable amount of time in the quadrant that previously housed the escape platform. These data suggest similar ability to retain memory of the location of the escape platform in KOs and WTs once the task has been learned. Estimated swim speed was also consistently comparable between KOs and WTs (*p*>.05, data not shown), suggesting that impaired swim speed was not a contributor to the KOs' delayed learning in the second version of the maze.

### Cognitive-Behavioral Flexibility

#### Y-maze

KOs made a significantly greater percentage of perseverative arm choices than did WTs (WT = 48.44±3.52, KOs = 63.53±4.05; *t* (31) = 2.81, *p* = .008), suggesting perseverative tendencies indicative of cognitive rigidity in the KOs. KOs and WTs did not differ significantly in latency to first arm choice (WT = 10.80±1.27, KOs = 10.00±1.26; *t* (31) = .493, *p* = .626) or in mean latency to arm choice across trials (WT = 54.79±7.83, KOs = 60.31±10.68; *t* (31) = .425, *p* = .674). In addition, significantly more KOs (25%) than WTs (5.5%) failed to make the minimum 4 consecutive arm choices for inclusion in the analysis (χ^2^ = 44.06, *p* = .000), suggesting behavioral reticence in the KOs in this paradigm.

#### Morris water maze

As reported above, KOs showed significantly higher mean latency to escape across trials compared to WTs in the second but not first version of the maze in both testing sequences (Figure 1).

### Exploratory Behavior and Anxiety

#### Open field

**Figure 2:**
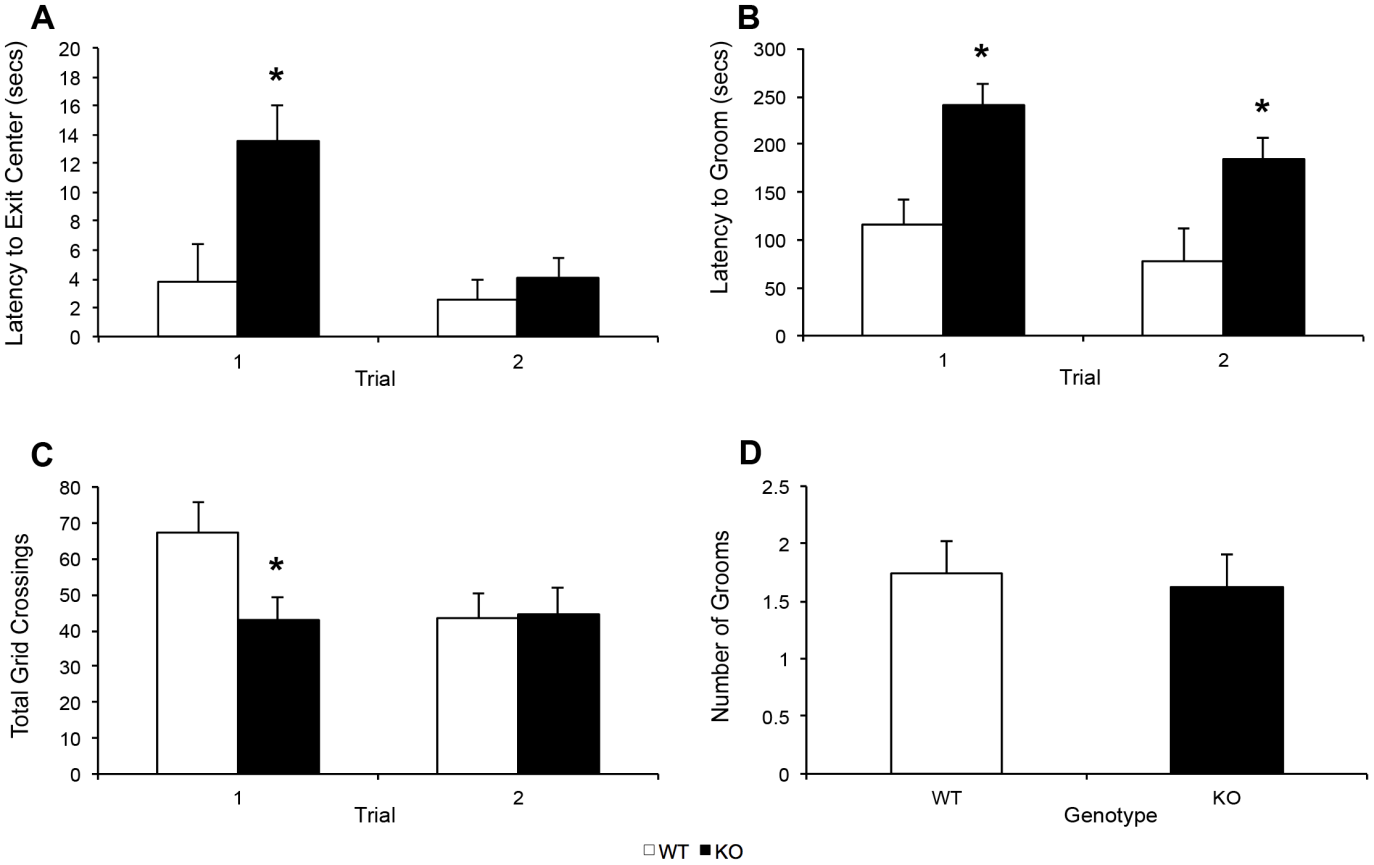
Open field behavior in PTN KOs and WTs. (a) Latency to exit the center grid, (b) latency to groom, (c) total grid crosses, and (d) number of grooms for KOs (n = 9) and WTs (n = 5). *p = <0.05.

KOs showed significantly greater latency to exit the center of the open field in the first but not second trial (*F* (1, 13) = 4.86, *p* = .046, Figure 2a). KOs also showed a significantly increased latency to groom in both trials (*F* (1, 14) = 4.35, *p* = .008, Figure 2b), and significantly fewer total grid crossings in the first but not second trial (*F* (1, 14) = 11.41, *p* = .005, Figure 2c). These findings are again suggestive of behavioral reticence in the KOs, but only early in their exposure to the paradigm when the context was novel. The genotypes did not differ significantly in overall number of grooms in the open field (*F* (1, 14) = .099, *p* = .758, Figure 2d), suggesting that delayed grooming in the KOs did not reflect a decreased tendency for motor stereotypy.

#### Elevated plus maze

**Figure 3:**
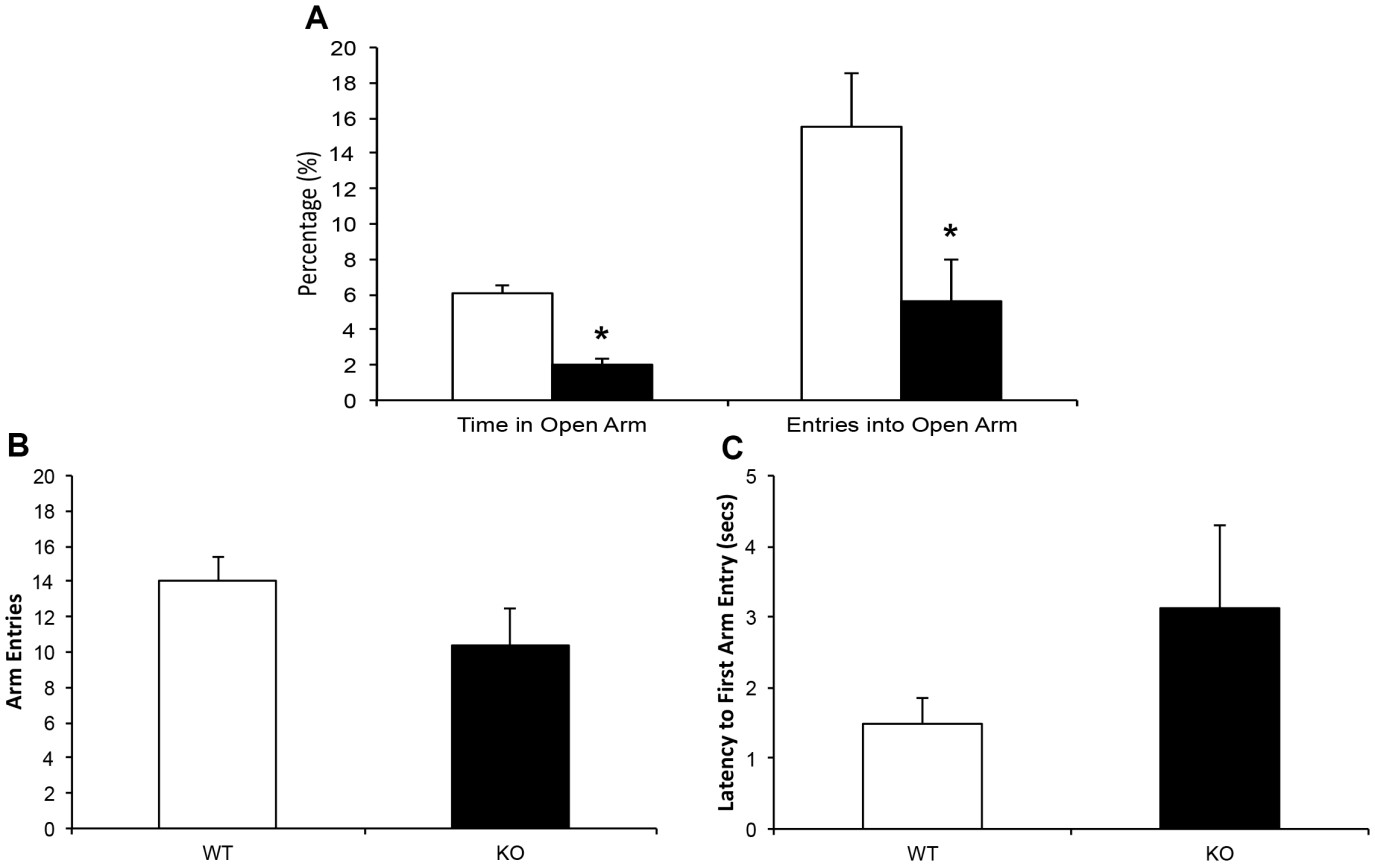
Elevated plus maze behavior in PTN KOs and WTs. (a) Percentage of time spent in, and entries into, open arms, (b) total number of arm entries, and (c) latency to first arm entry for KOs (n = 8) and WTs (n = 6). *p = <0.05.

KOs spent a significantly lower proportion of time in the open arms of the maze (*t* (12) = 2.38, *p* = .035) and made significantly fewer open arm entries than did WTs (*t* (12) = 2.44, *p* = .031, Figure 3a), suggesting increased anxiety-like behavior in the KOs. However, KOs did not differ significantly from WTs in total number of arm entries (*t* (12) = 1.31, *p* = .213, Figure 3b) or in latency to first arm entry (*t* (12) = 1.148, *p* = .273, Figure 3c), indicating no difference in overall motor activity between the genotypes.

### Social Behavior

#### Sociability test

**Figure 4:**
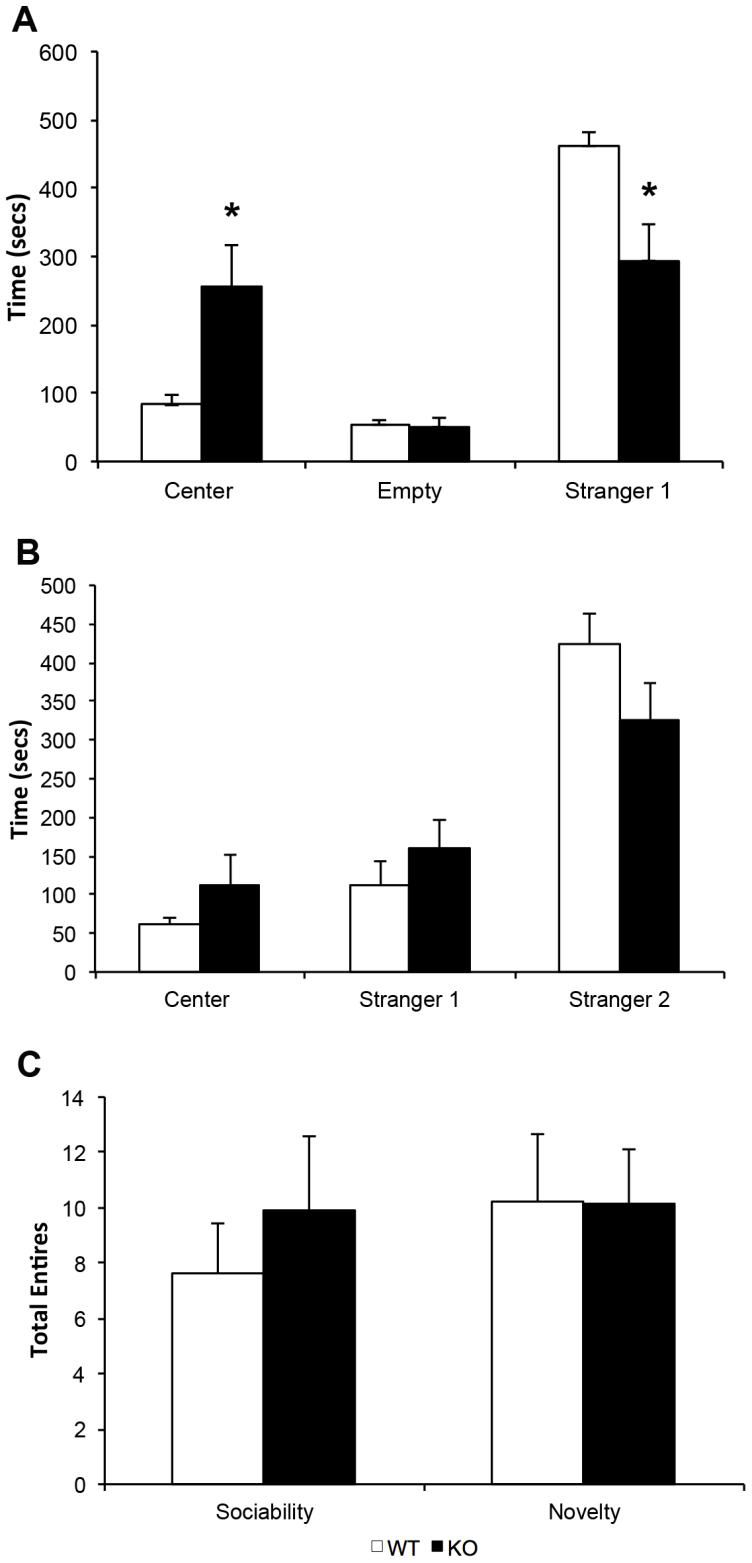
Social approach behavior in PTN KOs and WTs. (a) Time spent in the stranger 1 chamber, empty chamber, and center chamber during the sociability test; (b) time spent in the stranger 1 chamber, stranger 2 chamber, and center chamber during social novelty test; (c) total number of chamber entries made during the sociability and social novelty tests for KOs (n = 11) and WTs (n = 9). *p = <0.05.

KOs spent significantly less time in the stranger chamber (*t* (18) = 2.73, *p* = .011) and significantly more time in the center chamber than did WTs (*t* (18) = 2.45, *p* = .021), suggesting a decreased preference for social interaction with a novel conspecific in the KOs. The genotypes did not differ significantly in time spent in the empty chamber (*t* (18) = .233, *p* = .819, Figure 4a). KOs and WTs did not differ significantly in total number of chamber entries (*t* (18) = .707, *p* = .488, Figure 4c, left), again reflective of no difference in overall motor activity between the genotypes.

#### Social novelty test

KOs and WTs did not differ significantly in time spent in the stranger 1 chamber (*t* (18) = 1.37, *p* = .188), center chamber (*t* (18) = 1.40, *p* = .183), or stranger 2 chamber (*t* (18) = 1.88, *p* = .079, Figure 4b), but a statistical trend toward KOs spending less time in the stranger 2 chamber was observed. As during the sociability test, KOs and WTs did not differ significantly in total number of chamber entries (*t* (18) = .035, *p* = .973, Figure 4c, right).

### Neuroanatomical Evaluation

#### Lateral entorhinal cortical neurons

**Figure 5:**
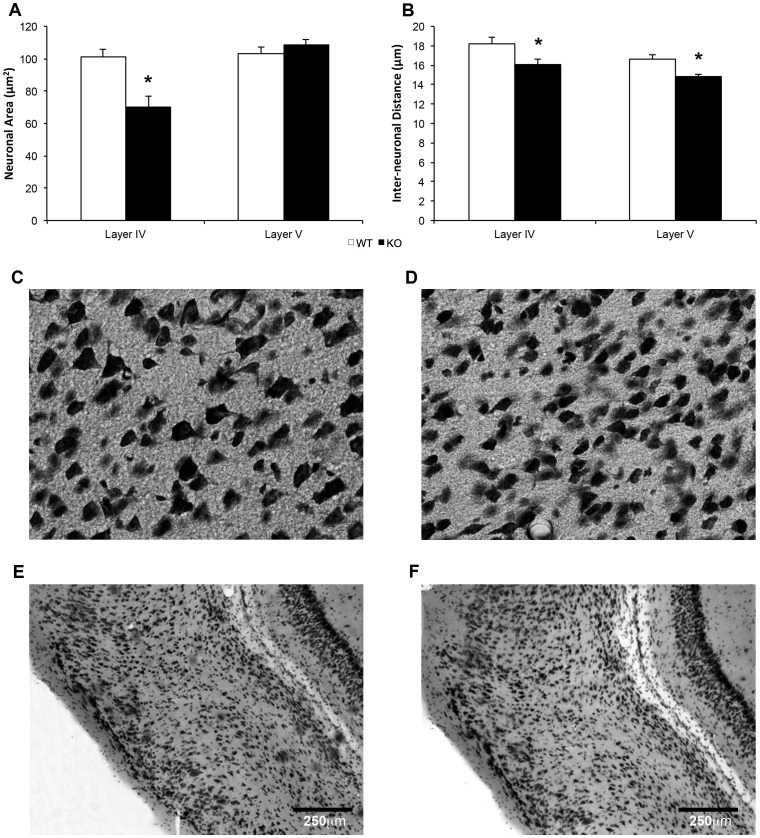
Entorhinal cortical cellular characteristics in PTN KOs and WTs. (a) Neuronal area and (b) interneuronal distance in layer IV and layer V of the entorhinal cortex in KOs (n = 9) and WTs (n = 10). Nissl-stained sections of layer IV of entorhinal cortical tissue in a (c) WT and (d) KO; and whole sections of entorhinal cortex in a (e) WT and (f) KO. Scale bar = 250 mm. *p = <0.05.

Neuronal area was significantly smaller in KOs than in WTs in layer IV (*t* (16) = 3.52, *p* = .003, Figure 5c & d) but not in layer V (*t* (17) = .125, *p* = .902; Figure 5a) of the lateral EC. Inter-neuronal distance in the EC was significantly smaller in KOs in both layers IV (*t* (17) = 2.24, *p* = .038) and V (*t* (17) = 3.22, *p* = .007; Figure 5b). EC thickness did not differ significantly between the genotypes (*t* (14) = 1.25, *p* = .232, data not shown). Together, these data suggest that the KOs' brains contain smaller, more densely-packed neurons in the lateral EC.

No significant correlations were observed between EC thickness and neuronal area in layer IV (*r* = .37, *p* = .151) or layer V (*r* = .29, *p* = .263) of the EC. However, a significant positive correlation was observed between EC thickness and inter-neuronal distance in layers IV (*r* = .732, *p* = .001) and V (*r* = .706, *p* = .002). Figure 5 shows representative whole sections of lateral EC in a WT (Figure 5e) and a KO (Figure 5f).

#### Entorhinal cortical vasculature

**Figure 6:**
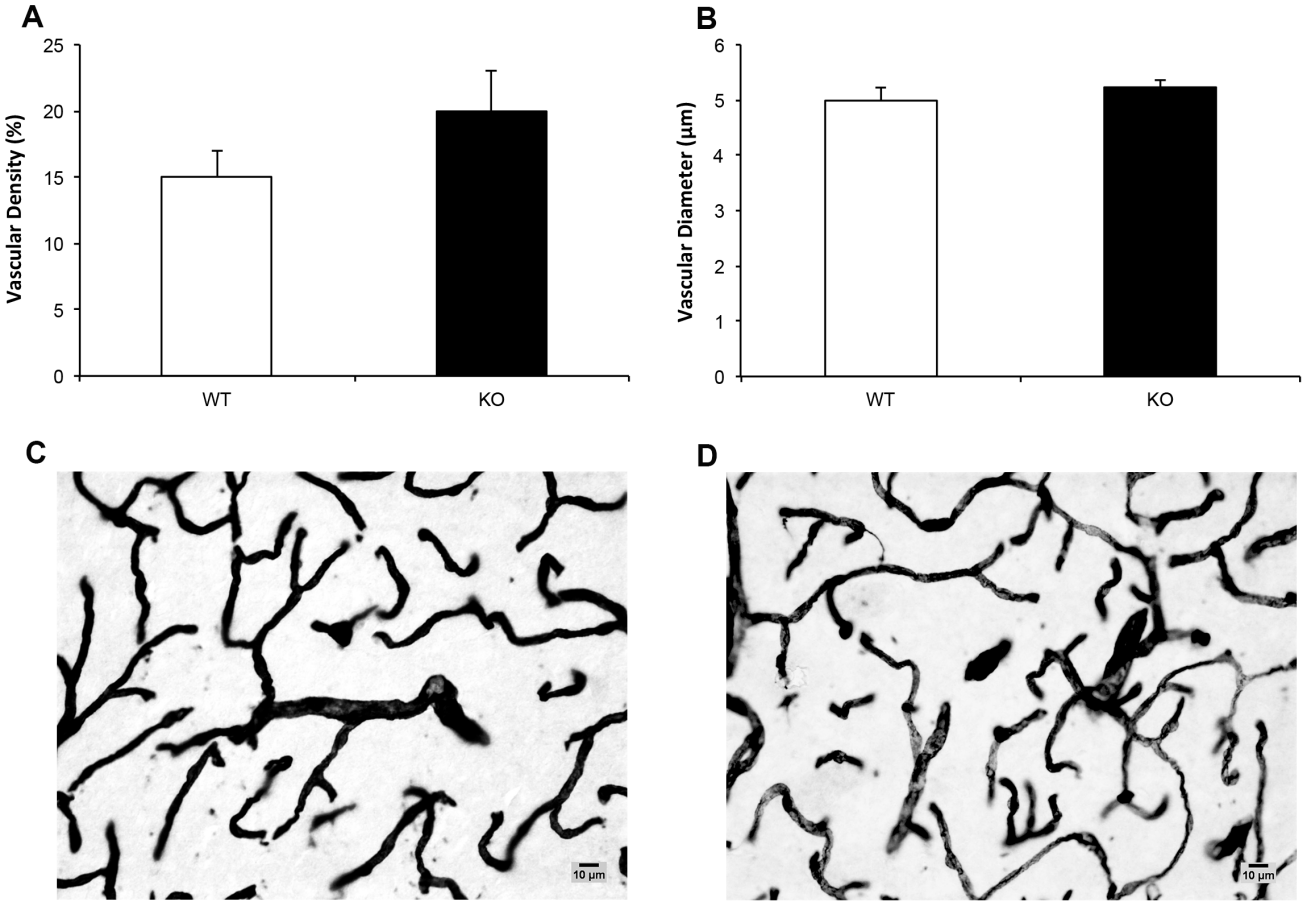
Entorhinal cortical vascular characteristics in PTN KOs and WTs. (a) Vascular density and (b) vascular diameter in entorhinal cortex for KOs (n = 9) and WTs (n = 10) with alpha collagen IV-stained sections of entorhinal cortical tissue from a (c) WT and (d) KO. Scale bar = 10 mm. *p = <0.05.

Vascular density in the lateral EC did not differ significantly between the genotypes (*t* (11) = .386, *p* = .707, Figure 6a, c & d), nor did vascular diameter (*t* (11) = .760, *p* = .463, Figure 6b, c & d).

## Discussion

### Summary

PTN KO mice were grossly normal but showed behavioral abnormalities suggestive of increased anxiety, behavioral reticence, and cognitive rigidity in addition to smaller, more densely-packed neurons in layer IV of the lateral EC, with no detectable abnormalities in overall cortical thickness or in vascular microanatomy.

### Learning and Memory

Water maze naïve PTN KOs showed no detectable impairments in learning or memory in the first version of the maze in which they were tested, whether that version was spatial or cued, suggesting normal initial spatial and associative learning. Pavlov et al. [25] reported reduced learning and retention in a spatial version of the water maze in PTN KOs, but these deficits were not striking. Indeed, del Olmo et al. [27] more recently reported findings indicative of enhanced retention of spatial information in the KOs. Collectively, our findings and those of previous work do not suggest that PTN KOs exhibit significant deficits in initial learning and memory.

### Cognitive-Behavioral Flexibility

In contrast to their normal performance in the first version of water maze in which they were tested, PTN KOs demonstrated slower but ultimately successful learning of the second version of the water maze in which they were tested, regardless of whether the second version was spatial or cued. Given no evidence of significant learning and memory deficits, these data might suggest that the KOs were less able to adapt to the change in task demands between the different versions of the water maze and, therefore, that the KOs showed a tendency for behavioral and/or cognitive rigidity. In support of this hypothesis, the KOs showed significantly less spontaneous alternation in the Y-Maze in favor of perseverative responding indicative of behavioral and/or cognitive inflexibility.

Previous literature indicates that PTN KOs have a reduced threshold for induction of hippocampal LTP *ex vivo* [22], [24]. Therefore, the absence of PTN might lead to an enhancement of synaptic strength in the KOs. This enhanced strength of neural circuits in the PTN KO brain might mean that those circuits are more difficult to remodel. Therefore, the first escape strategy the KOs learned in the water maze, and the specific arm choices KOs made in the Y-maze, might have been synaptically “overlearned.” This idea is further supported by previous findings of prolonged maintenance of recognition memory [27] and habit-driven behavior [28] in the KOs.

Other possible mechanisms for cognitive inflexibility in the KOs include heightened vulnerability to LTP saturation, which can impair spatial learning [37], enhanced heterosynaptic long-term depression [38], [39], and a lowered threshold for the formation of stimulus-reward associations. Future research should seek to determine the specific synaptic abnormalities in PTN KOs that underlie their apparent cognitive-behavioral inflexibility.

### Exploratory Behavior and Anxiety

In the elevated plus maze, PTN KOs spent significantly more time in the closed arms of the maze and made significantly fewer entries into its open arms, suggesting increased anxiety consistent with results reported by Pavlov et al. [25]. In the open field, PTN KOs demonstrated a significantly increased latency to exit the center in the first but not second trial. Because mice tend to avoid open, brightly lit areas, increased latency to exit the center of the open field is often considered an indicator of reduced anxiety [31], [32]. In contrast, the competing behavior of anxiety-induced freezing would lengthen latency to move, complicating interpretation of our finding. However, because the KOs made significantly fewer grid crossings in the first but not the second open field trial, increased anxiety in response to a novel environment is a more likely explanation than decreased anxiety. KOs' behavior in the open field likely reflected increased anxiety in the form of freezing given that we observed no evidence of reduced exploration in the KOs. Indeed, the KOs also showed an overall increased latency to groom in the open field, which has previously been characterized as hypoactivity secondary to anxiety [40].

Disruption of either PTN or the homologous protein midkine has been associated with heightened anxiety in mice [25], [41]. Indeed, many molecules responsible for developmental modulation of learning and memory systems, such as PTN, have been implicated in the activity of systems subserving fear-based behavior [42], [43]. However, recent work by Gramage et al. [44] suggests normal elevated plus maze behavior in PTN KOs. Disagreement between our findings and those of Gramage et al. might be due to differences in background strain between our cohorts or the fact that Gramage et al.'s protocol involved repeated exposure to the Y-maze up to 72 hours before elevated plus maze testing, constituting recent test experience that might have mitigated animals' sensitivity to the anxiety-provoking conditions of the test. Future studies of PTN KO behavior could focus mostly on tests of anxiety and incorporate longer inter-test intervals to potentially resolve the conflicting findings of previous studies. Nevertheless, our findings that PTN KOs exhibit heightened anxiety, perhaps particularly in novel contexts, suggests that future assessment of PTN KOs' behavior should involve use of less anxiogenic paradigms when possible and when indicated by the hypotheses under study, e.g. the T-maze [45] and attention set-shifting tasks [46] instead of or in addition to paradigms like the Morris water maze, which are known to evoke anxiety [30].

### Social Behavior

In the three-chambered social approach apparatus, KOs spent significantly less time in the chamber containing a novel conspecific and significantly more time in the empty and more familiar center chamber. Following the introduction of a second novel conspecific 15 minutes into testing, however, KOs did not differ significantly from WTs in time spent in the center or in the chamber housing the first novel conspecific. KOs spent less time in the chamber housing the more novel, second conspecific than did WTs, although this finding did not achieve statistical significance. Anecdotal observations of the KOs' home cage behavior toward cage-mates suggested qualitatively normal social interactions. Therefore, the KOs' behavior in the social approach paradigm might provide additional evidence of behavioral reticence in the presence of novelty rather than a purely social deficit.

### Entorhinal Cortical Neurons

PTN KO brains showed a significant, mean 30% decrease in neuronal area in layer IV and significantly decreased inter-neuronal distance in layers IV and V of the EC, but overall EC thickness was normal. Approximately 82% of the variance in EC thickness was accounted for by neuronal area and inter-neuronal distance in layers IV and V, suggesting that different but proportional neuronal area and inter-neuronal distance irregularities might exist in other cortical layers or that abnormalities exist exclusively in layers IV and V. Comparable overall EC thickness between KOs and WTs suggests the latter and indicates the presence of subtle, layer-specific neuronal irregularities not detectable via gross measures of overall cortical morphology. Collectively, these data are consistent with the observation of grossly normal brain size [24] but increased neuronal density in the frontal and parietal cortices of PTN KOs [9].

PTN has neurotrophic and neuroprotective functions both during development [9] and in pathological states [16], [18]. Reduced neuronal area in layer IV of the EC might reflect loss of these trophic and/or protective effects in the KOs. Further, neuronal hyperdensity in the lateral EC in PTN KOs could be caused by early hyperproliferation and hypodifferentiation of these cells, which would result in large numbers of small neurons. However, KO brains have not been reported to show increased neuronal numbers in frontal or parietal cortices, where neuronal density increases have been reported [9], suggesting that loss of PTN's pro-migratory effects might better explain our findings.

The EC maintains extensive connectivity to uni- and multi-modal sensory cortices as well as hippocampus [47], [48] and amygdala [49]–[51]. Accordingly, the EC has been implicated in declarative memory [52], [53], processing of affectively relevant stimuli, and behavioral modulation based on emotional valance of stimuli [54]. Primates with EC lesions make overall negative evaluations of affective stimuli, resulting in decreased approach and increased defensive behaviors [51]. In addition, anxiety-induced hyperalgesia is associated with entorhinal-hippocampal circuit activation in humans [55].

Abnormal neuronal size and packing density in the lateral EC of PTN KOs might therefore contribute to their heightened anxiety and neophobia. Indeed, assemblages of undersized neurons might be less able to relay signals across relatively long neural distances, causing disruptions in neural integration that would likely be associated with absent, delayed, and/or inappropriate behavioral responses to stimuli [56], [57]. More research is needed to better elucidate the characteristics of these circuits in PTN KOs.

### Entorhinal Cortex Vasculature

EC vasculature did not differ significantly between KOs and WTs, and no gross vascular pathology (e.g. tortuosity) was observed in the KOs. These data were somewhat unexpected given PTN's reported role in developmental angiogenesis [13], [14], the association between neurogenesis and vasculogenesis [58], [59], and the hypothesized impact of vascular status on progenitor cell proliferation and neuronal differentiation in both developmental and pathological states [60]. The homologous protein midkine has many of the same development effects as PTN [61], suggesting that compensatory effects of midkine are a possible explanation for the preservation of vascular development in the PTN KO brain. Alternatively, EC neuronal abnormalities in the KOs could be driven by subtle, transitory vascular defects or be independent of cerebral vascular status.

### Limitations

Each of the behavioral tests used in this study were conducted in at least 2 of 4 cohorts. Therefore, no animals were exposed to every test in the battery, raising the possibility that differing exposure to behavioral tests could have impacted our findings. Using 129B6 and C57BL/6J mouse strains, McIllwain et al. [62] conducted experiments to determine whether behavioral test performance was affected by prior exposure to testing and found that mice with prior exposure were comparable to naïve mice on levels of anxiety-like behavior (center-to-total distance ratio and number of crosses in the light–dark apparatus) in the open field as well as in learning in the Morris water maze. These findings suggest that previous test experience need not strongly influence learning or anxiety-related behavior and, therefore, that testing effects probably did not significantly impact our findings.

Test order was counterbalanced among our cohorts to ensure that prior testing experience could not have systematically contributed to our findings, but we cannot eliminate the possibility that test order had some impact. Very few differences in behavioral test performance as a function of test order have been reported in inbred strains [62], but no data regarding the effects of previous exposure to behavioral testing, test order, or different inter-test intervals on the behavior of PTN KOs are currently available. These issues could be addressed in future studies.

### Conclusion

Our findings suggest that PTN KO mice show an abnormal cognitive-behavioral phenotype characterized by perseverative tendencies indicative of cognitive rigidity; heightened anxiety; behavioral reticence in novel spatial or social contexts suggestive of neophobia; and microscopic neuroanatomical abnormalities including decreased neuronal area in layer IV, and neuronal hyperdensity in layers IV and V, of the lateral EC.

Interestingly, cognitive rigidity, anxiety, and neophobia are cardinal features of autism spectrum disorder (ASD) [63], and accumulating evidence suggests that decreased neuronal area and neuronal hyperdensity are present in the limbic structures of affected individuals [64]. Further study of PTN KO mice might therefore have implications for the development of animal models of ASD.
